# Supplementary material for: Increased Resection at DSBs in G2-Phase Is a Unique Phenotype Associated with DNA-PKcs Defects That Is Not Shared by Other Factors of c-NHEJ
Source: Cells. 2022 Jul 2;11(13):2099. doi: 10.3390/cells11132099 (PMC9265841; doi:10.3390/cells11132099)
Supplement: Supplementary file 1 [file cells-11-02099-s001.zip › cells-1778064-supplementary.pdf]

Supplementary Information

Increased resection at DSBs in G_2-phase is a unique phenotype associated with DNA-PKcs defects that is not shared by other factors of c-NHEJ

Huaping Xiao Fanghua Li, Emil Mladenov, Aashish Soni, Veronika Mladenova, Bing Pan, Rositsa Dueva, Martin Stuschke, Beate Timmermann and George Iliakis

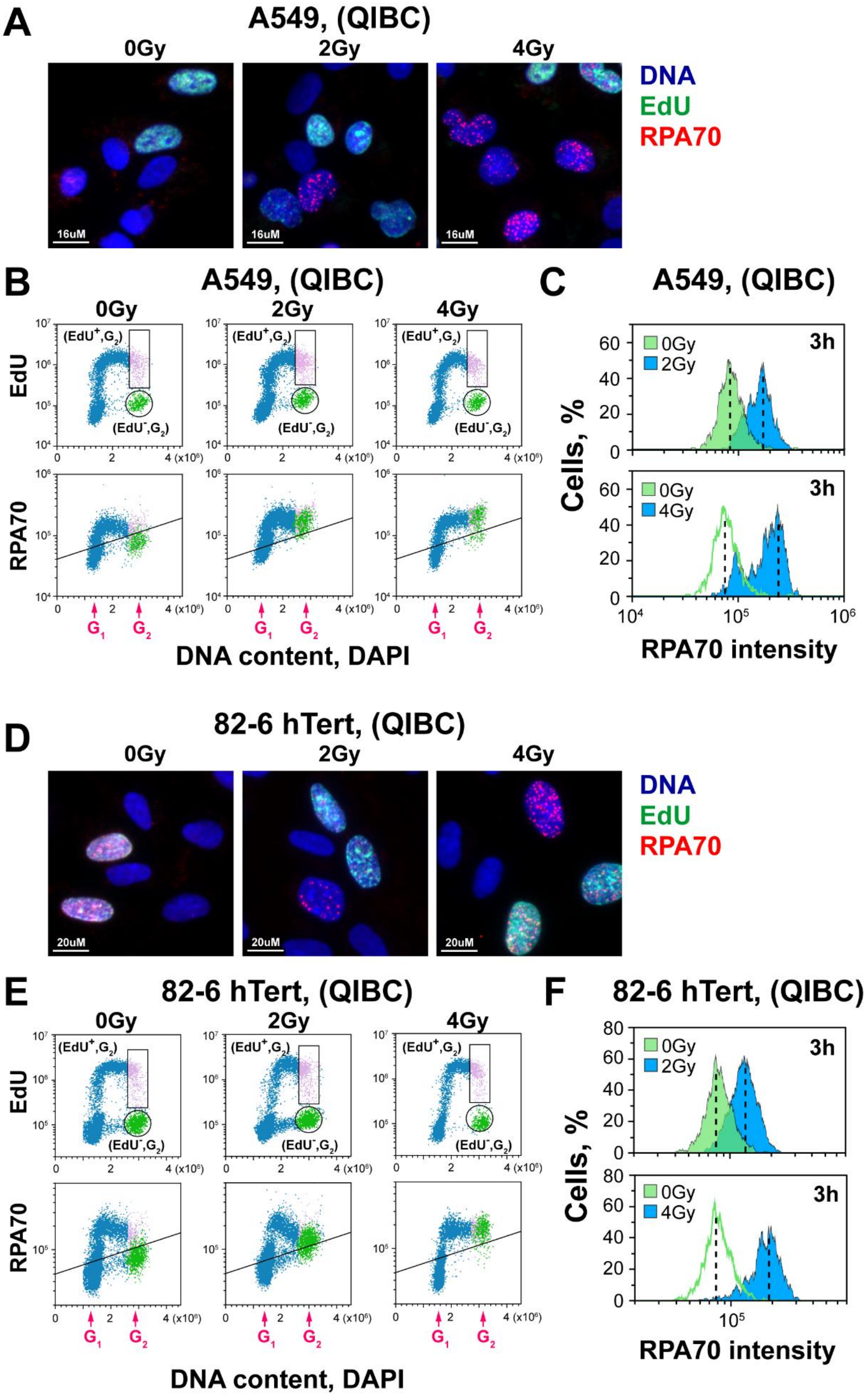
A
A549, (QIBC)
0Gy
2Gy
4Gy
DNA
EdU
RPA70
16uM
B
A549, (QIBC)
EdU
RPA70
(EdU+,G2)
(EdU-,G2)
G1
G2
DNA content, DAPI
C
A549, (QIBC)
0Gy
2Gy
4Gy
3h
Cells, %
RPA70 intensity
D
82-6 hTert, (QIBC)
0Gy
2Gy
4Gy
DNA
EdU
RPA70
20uM
E
82-6 hTert, (QIBC)
EdU
RPA70
(EdU+,G2)
(EdU-,G2)
G1
G2
DNA content, DAPI
F
82-6 hTert, (QIBC)
0Gy
2Gy
4Gy
3h
Cells, %
RPA70 intensity

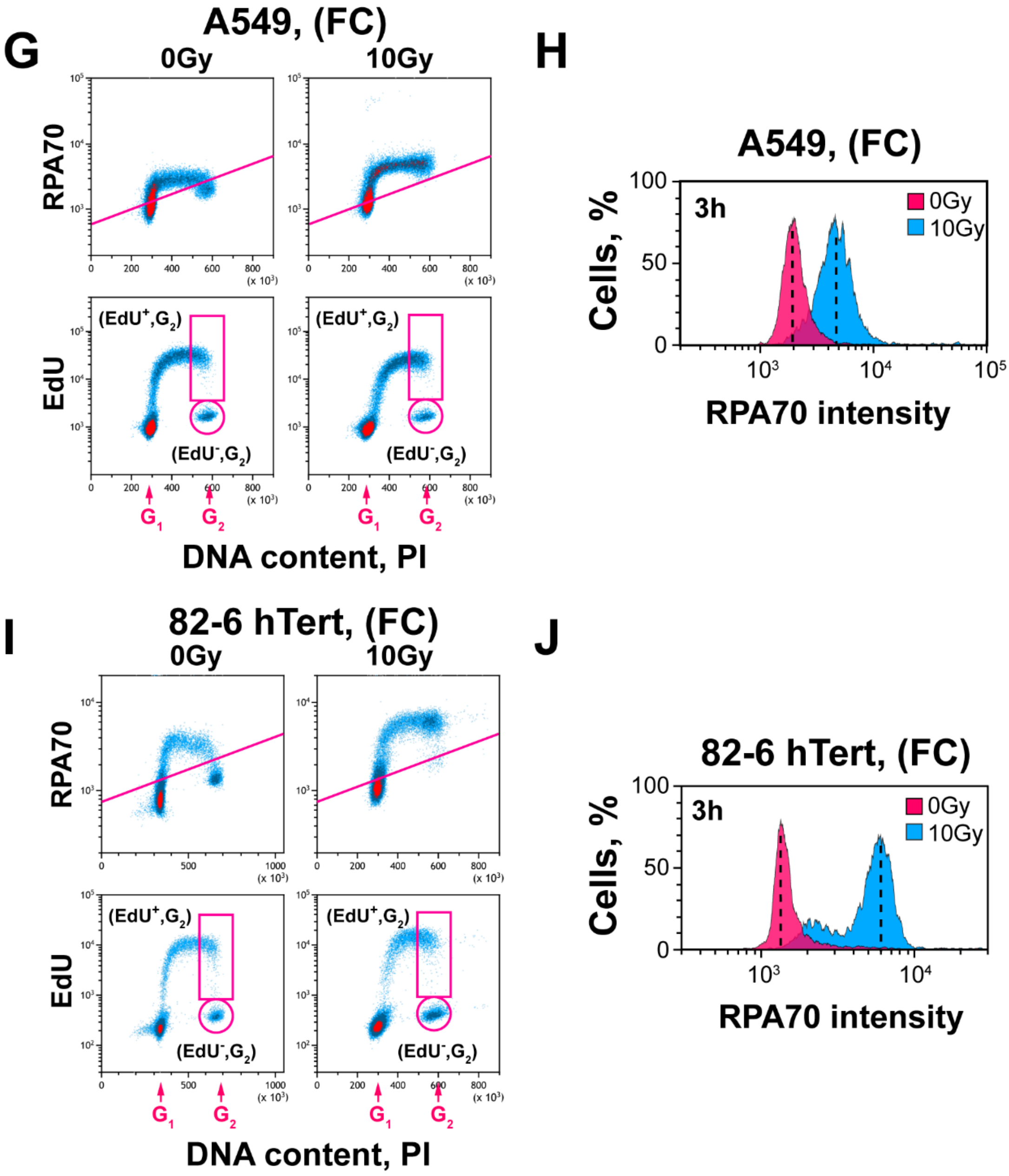

Figure S1. *Outline of methodology to measure resection in G$_2$-phase.* A) Representative AxioScan Z1 immunofluorescent images, obtained during QIBC analysis of A549 cells irradiated with 2 and 4 Gy X-rays. DNA was counterstained with DAPI and DAPI intensity (blue) was assessed to determine the progression of cells through the cell cycle. EdU intensity signal (green) was evaluated to determine whether cells were irradiated in S-phase, while the accumulation of RPA70 (red) onto chromatin was an indication for ongoing DNA end resection. B) Dot plots of RPA70, EdU and DAPI intensities obtained by QIBC analysis. The gates utilized to specifically select cells in different cell cycle phases

are also depicted. EdU positive (EdU^+, violet) or EdU negative (EdU^-, green) cells, represent the cells irradiated, respectively, in S- or G_2-phase of the cell cycle. Counterstaining with DAPI allows the determination of DNA content, which reflects the cell cycle phase during analysis. For our experiments, we have selected cells irradiated in G_2-phase and analyzed in the G_2-phase (EdU^-, G_2-cells, green). C) The RPA70 intensity signals in the selected gates (EdU^-, G_2-cells) are shown as histogram plots. DNA end-resection after IR is visualized by the increase in the signal intensity between irradiated and non-irradiated cells. The ratio of the means between the two signal intensities is used as a parameter to quantitate resection in the experiments shown here. D, E and F) As in panels A, B and C for 82-6 hTert cells. G) Three parametric flow cytometry analysis of RPA70 signal intensity in A549 cells, showing the gates utilized to select cells irradiated in the G_2-phase and analyzed in G_2-phase of the cell cycle (EdU^-, G_2-cells). H) The RPA70 intensity signals in the selected gates (EdU^-, G_2-cells) are plotted as histogram plots. Other details as in Fig. S1A-C. I) As in panel G, for 82-6 hTert cells. J) As in panel H, for 82-6 hTert cells.

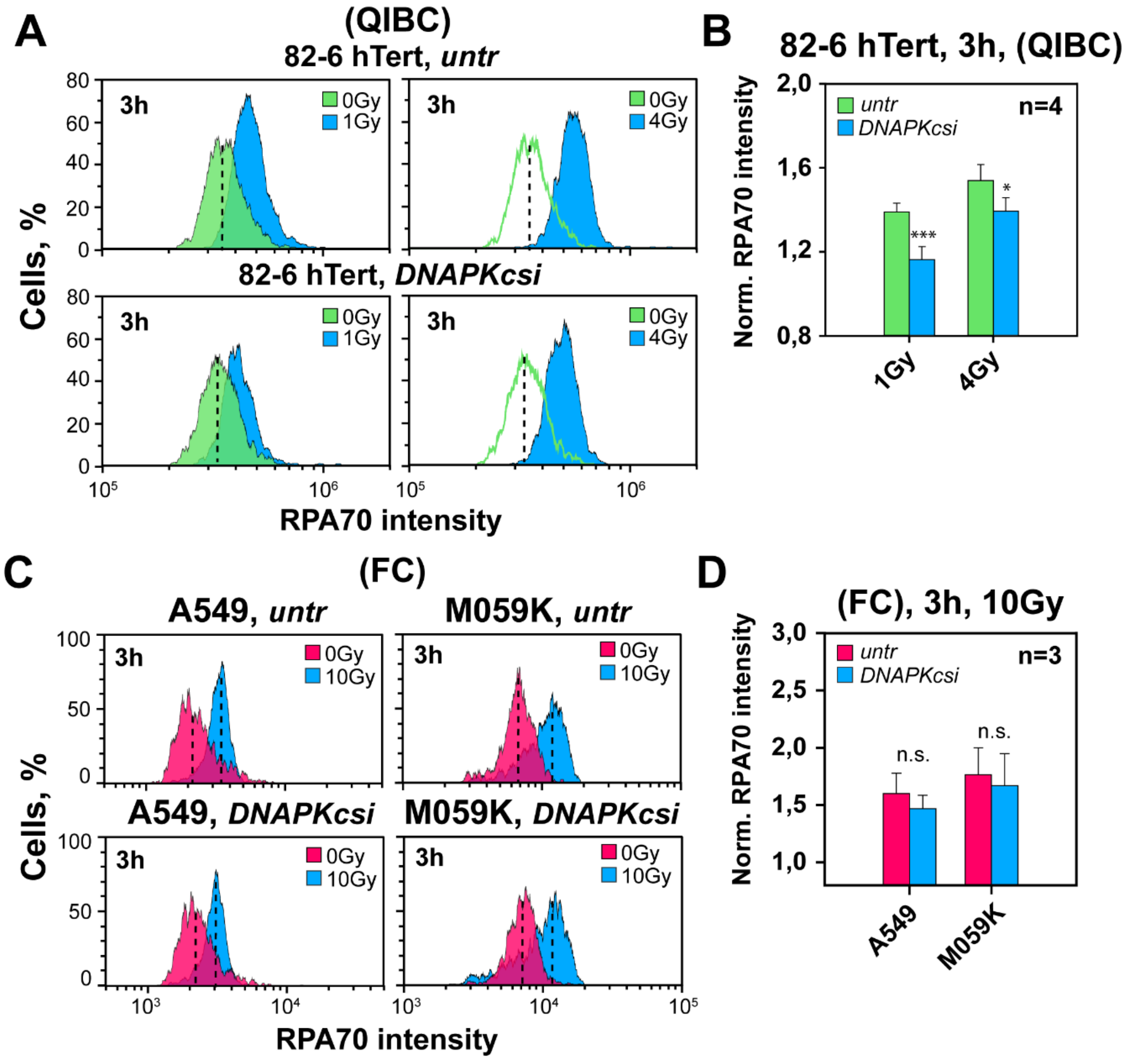

Figure S2. *Resection analysis in DNA-PKcs deficient cells.* A) QIBC analysis of RPA70 signal at DSBs in 82-6 hTert cells treated or not with *DNA-PKcsi (NU7441),* 3 h after exposure to 1 Gy or 4 Gy. Other details as in Fig. 1B. B) Bar plot showing the normalized RPA70 signal intensity from three experiments, obtained as shown in panel A. C) FC-based analysis of resection in wild-type A549 and M059K cells, treated or not with *DNA-PKcsi*. DNA end-resection is measured 3 h after exposure to 10 Gy (other details as in Fig. 1D). D) Bar plot showing the normalized RPA70 signal intensity from three experiments, obtained as shown in panel C.

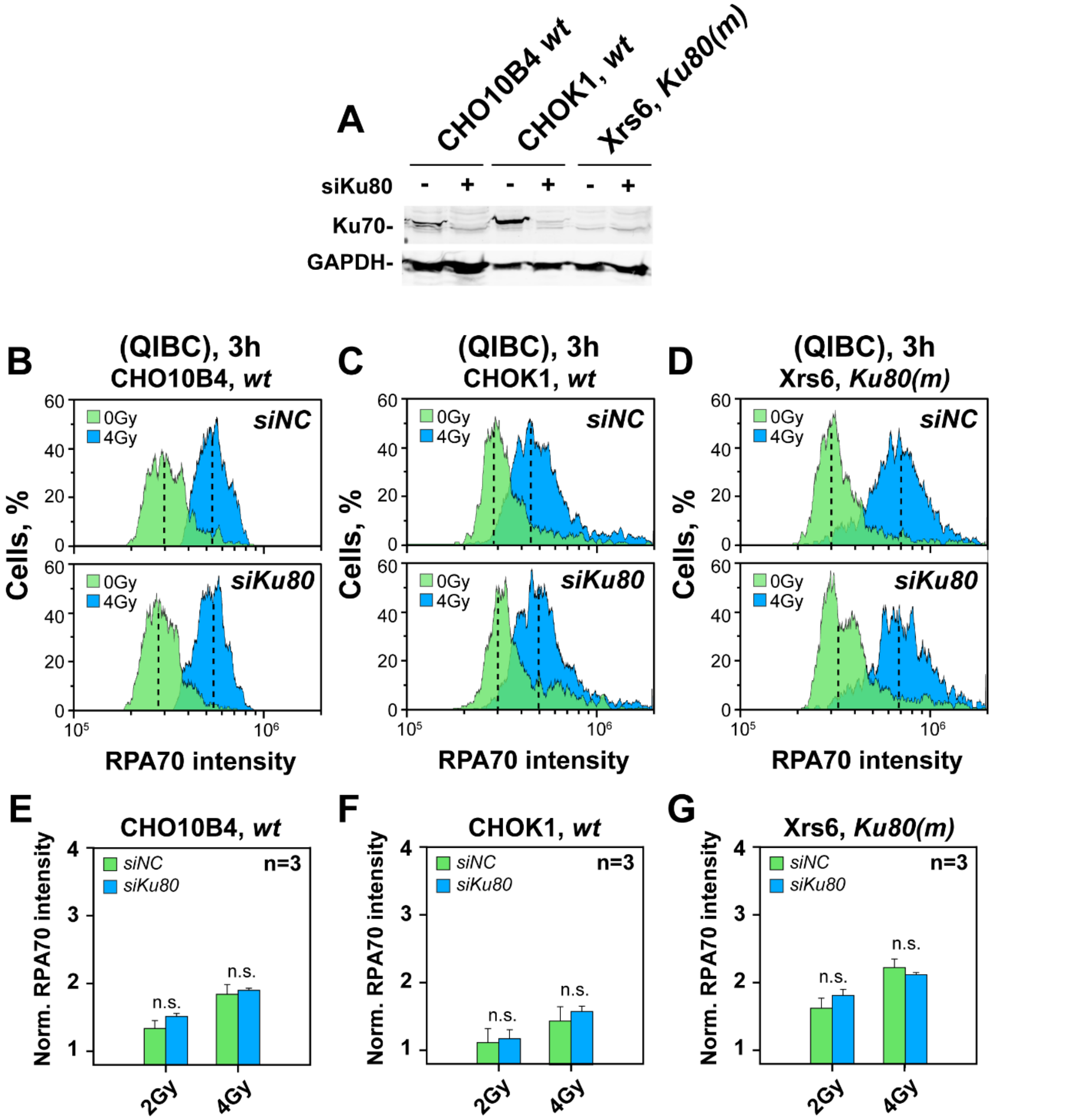

Figure S3. *Resection analysis in CHO cells after knockdown of Ku.* A) Western blot analysis showing the level of Ku70 in CHO10B4, CHOK1 and Xrs6 cells 24 h after transfection with a siRNA against Ku80, or with a non-specific control siRNA. KU70 serves here as a proxy for KU80, as the anti-KU80 antibody we used failed to recognize the hamster protein. B) Representative histograms of RPA70 intensity in mock transfected and Ku depleted CHO10B4(wt) cells, obtained by QIBC analysis 3 h after exposure to 2 or 4 Gy. C) As in panel B, for CHOK1(wt) cells. D) As in panel B, or the Ku80 mutant. E) Quantitative analysis of RPA70 signal in CHO10B4(wt) cells. F) As in panel E, but for CHOK1(wt) cells. G) As in panel E, for Xrs6 cells. The results represent the mean and SE from 3 independent determinations.

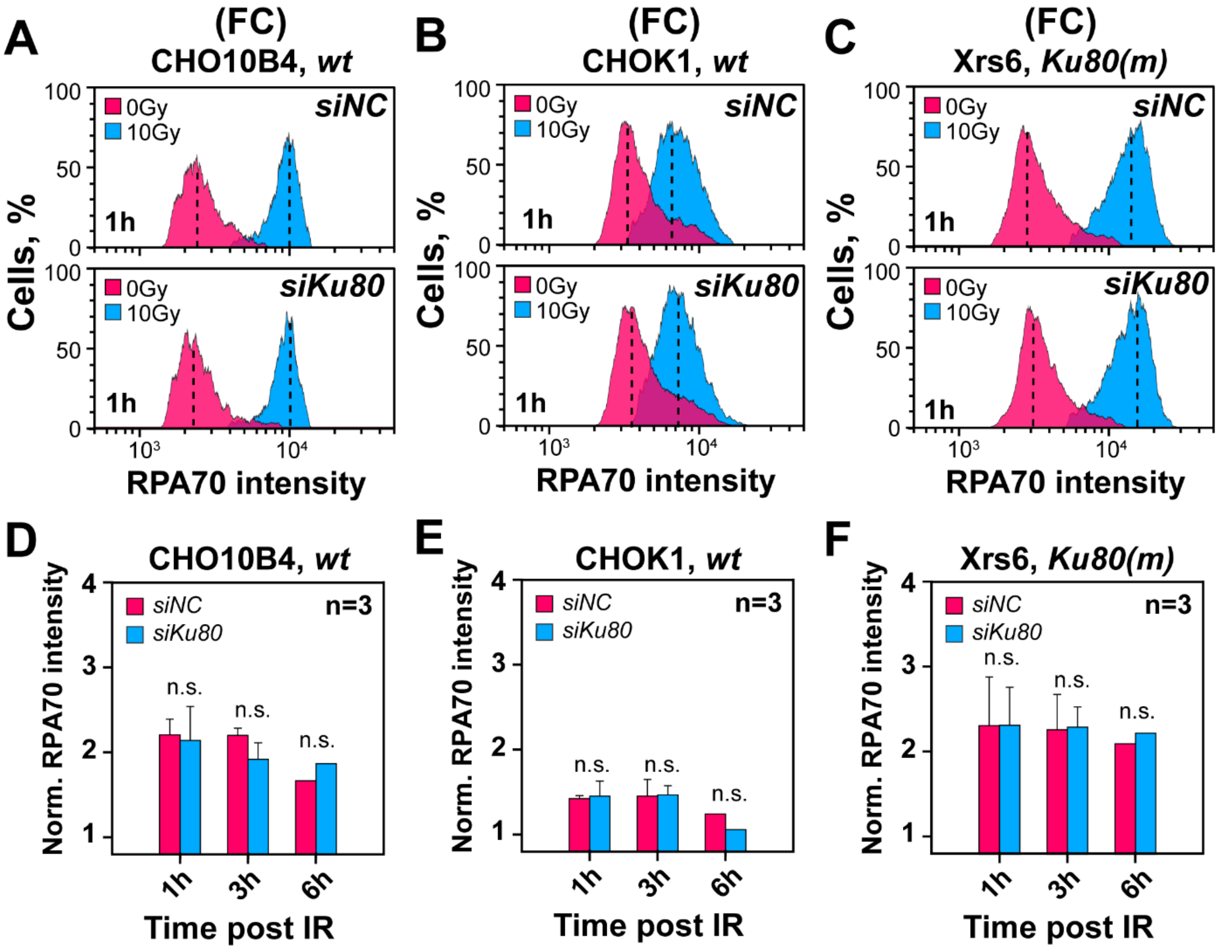

Figure S4. *Resection after Ku knockdown in CHO cell lines*. A) Representative histograms of RPA70 intensity in mock transfected and Ku depleted CHO10B4(wt) cells, obtained by FC analysis 1 h after exposure to 10 Gy. B) As in panel A, for CHOK1 cells. C) As in panel A, for Xrs6 cells. D) Quantitative analysis of RPA70 signal in CHO10B4 cells. E) As in panel D, for CHOK1 cells. F) As in panel D, for Xrs6 cells. The results represent the mean and SE from 3 independent determinations.

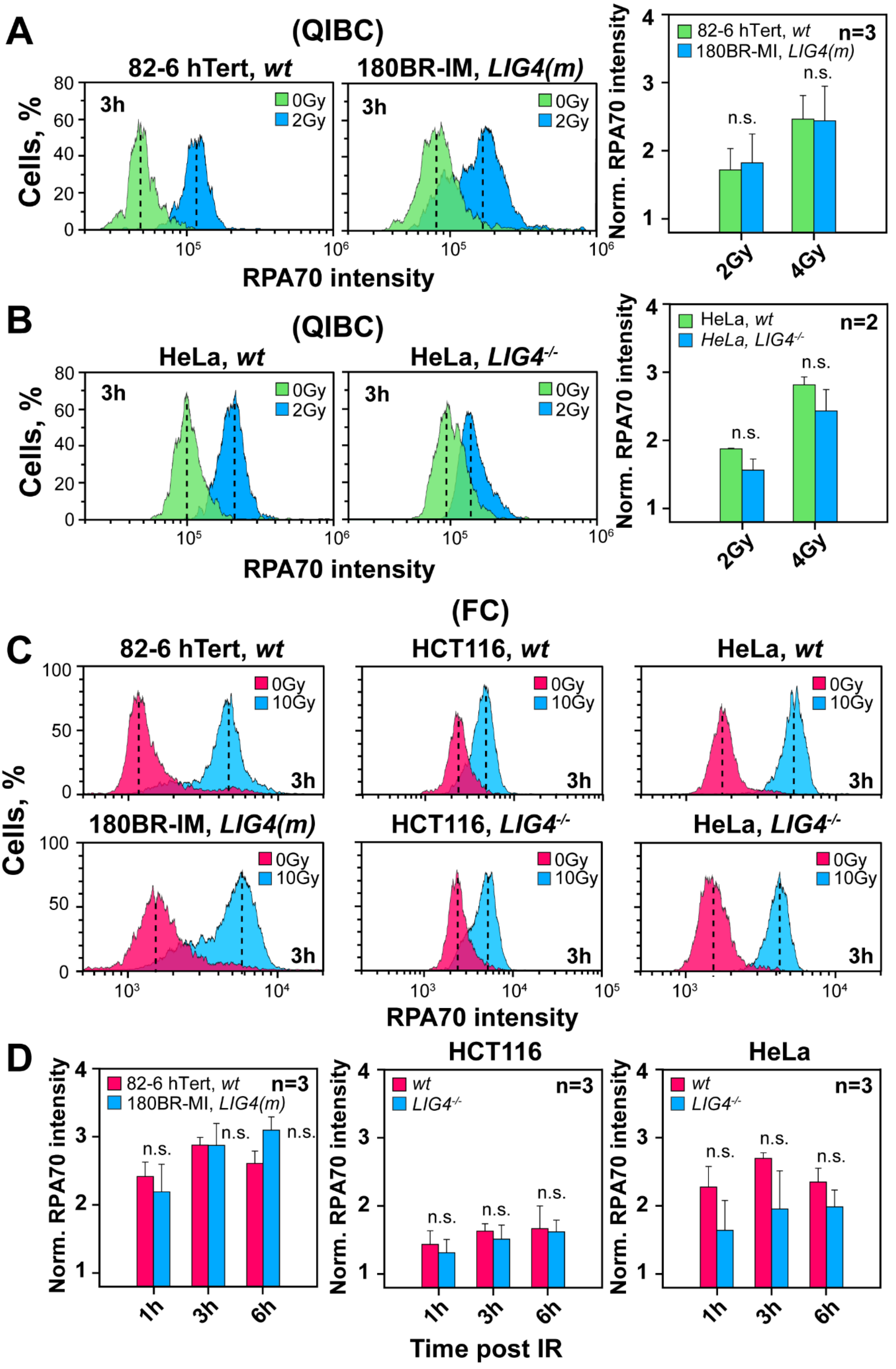
A
(QIBC)
82-6 hTert, wt
180BR-IM, LIG4(m)
3h
0Gy
2Gy
Cells, %
RPA70 intensity
Norm. RPA70 intensity
82-6 hTert, wt
180BR-MI, LIG4(m)
n=3
n.s.
2Gy
4Gy
B
(QIBC)
HeLa, wt
HeLa, LIG4-/-
n=2
C
(FC)
82-6 hTert, wt
HCT116, wt
HeLa, wt
180BR-IM, LIG4(m)
HCT116, LIG4-/-
HeLa, LIG4-/-
0Gy
10Gy
3h
D
HCT116
HeLa
wt
LIG4-/-
1h
3h
6h
Time post IR

Figure S5. *Resection analysis is LIG4 deficient cells.* A) QIBC analysis of RPA70 in wt, 82-6 hTert and LIG4 deficient 180BR-M human cell lines. The bar plot is showing the normalized RPA70 signal intensity from three independent determinations. B) As in panel A for LIG4 proficient and deficient HeLa cells. C) FC-based analysis of RPA70 signal intensity in human cell lines deficient in LIG4. Other details as in Figure 2B. D) Quantification of RPA70 signal from three experiments such as those shown in panel A.

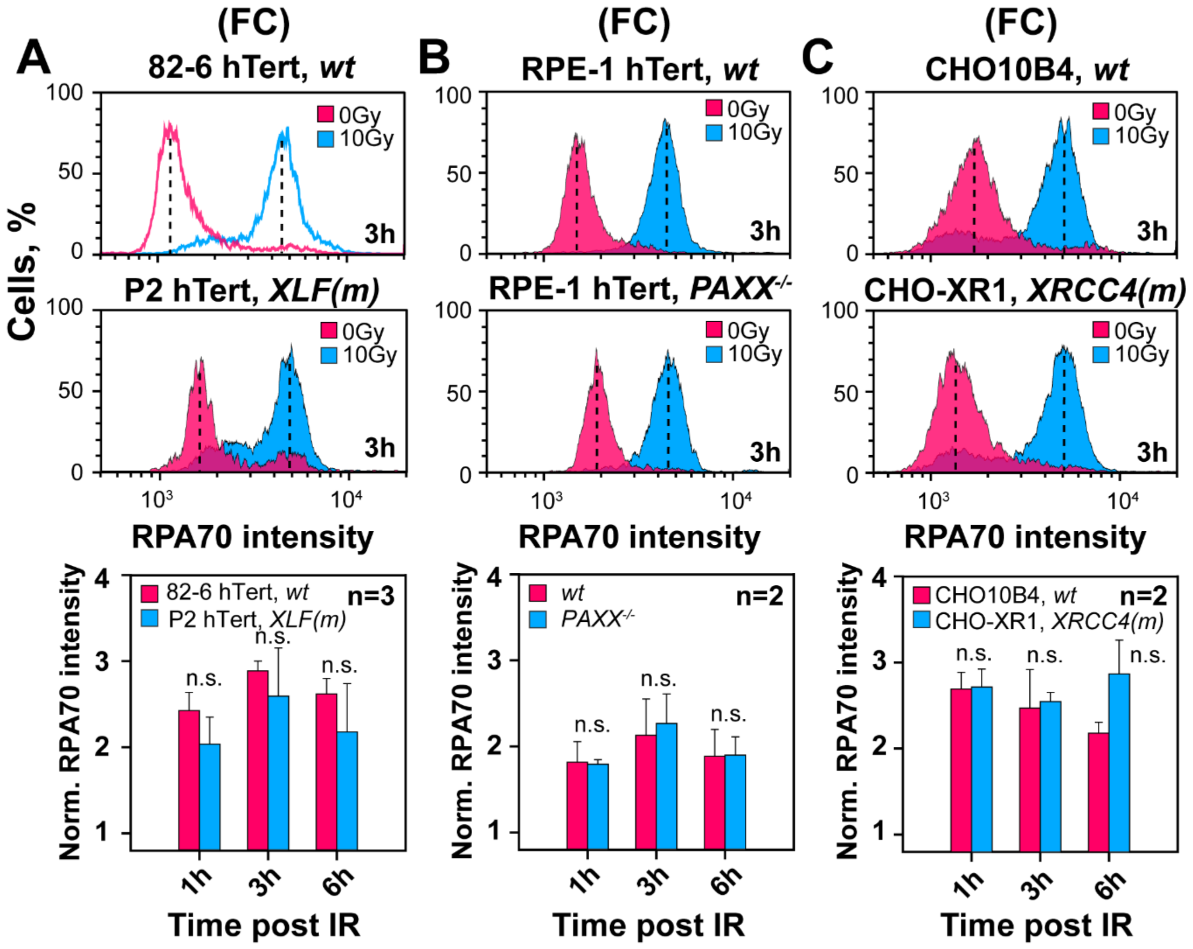

Figure S6. *FC-based analysis of resection in c-NHEJ deficient cells.* A) Resection in *XLF*$^{-/-}$ cells. B) Resection in *PAXX*$^{-/-}$ cells. C) Resection in *XRCC4*$^{-/-}$ cells. All cell lines were exposed to 10 Gy and analyzed at 1 h, 3 h, and 6 h after irradiation. The experiments are repeated thrice and the bar graphs at the bottom show the combined results. Plotted are the mean and SE from three independent experiments.

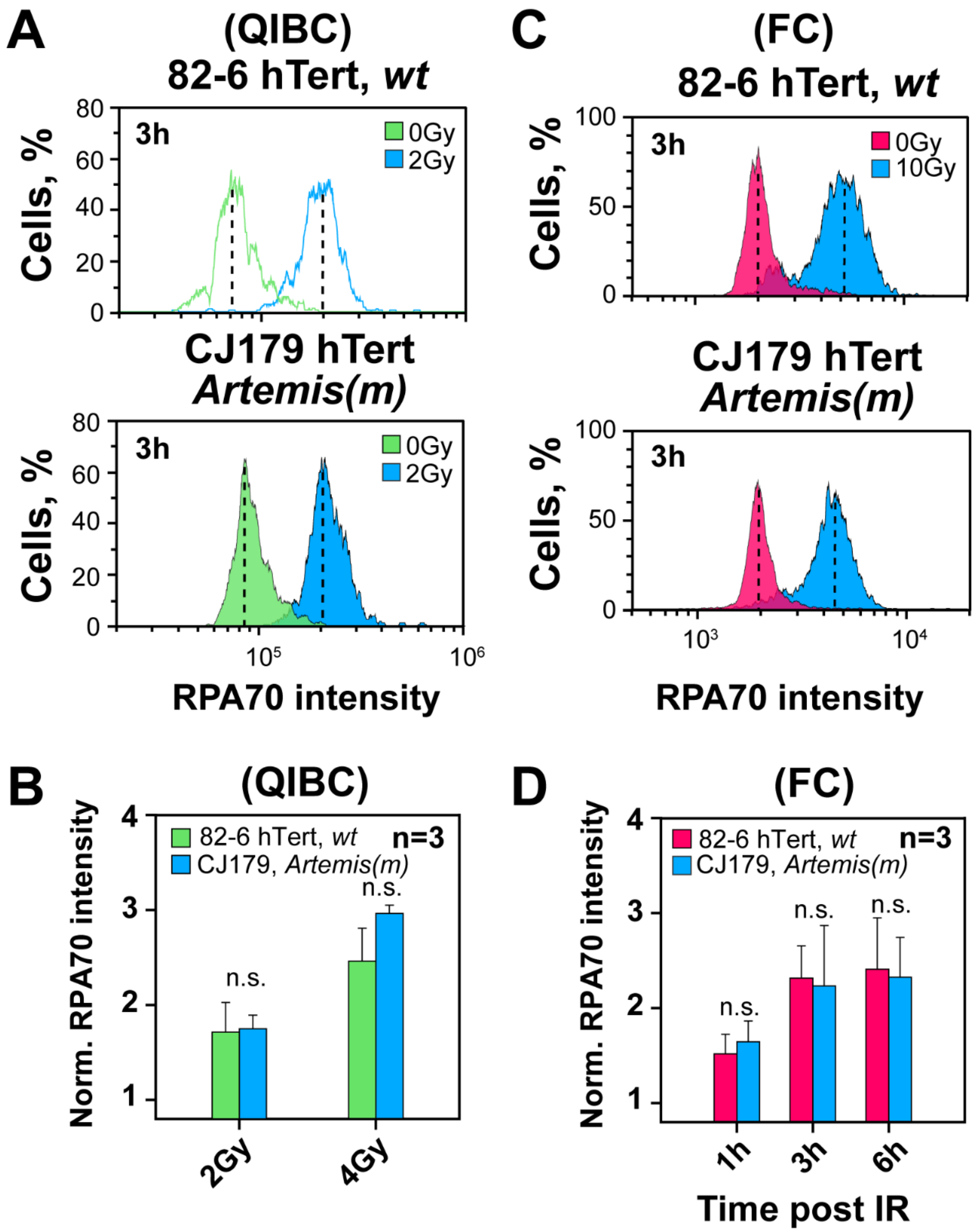

Figure S7. *Resection analysis in Artemis$^{-/-}$ cells.* A) RPA70 signal intensity determined using QIBC 3 h after exposure to 2 Gy or 4 Gy of IR (only data for 2 Gy are shown). B) Quantification of RPA70 signal intensity determined as shown in panel A. C) As in panel A, using FC after exposure to 10 Gy. D) As in panel B, for experiments shown in panel C. Plotted are the mean and SE from three independent experiments.

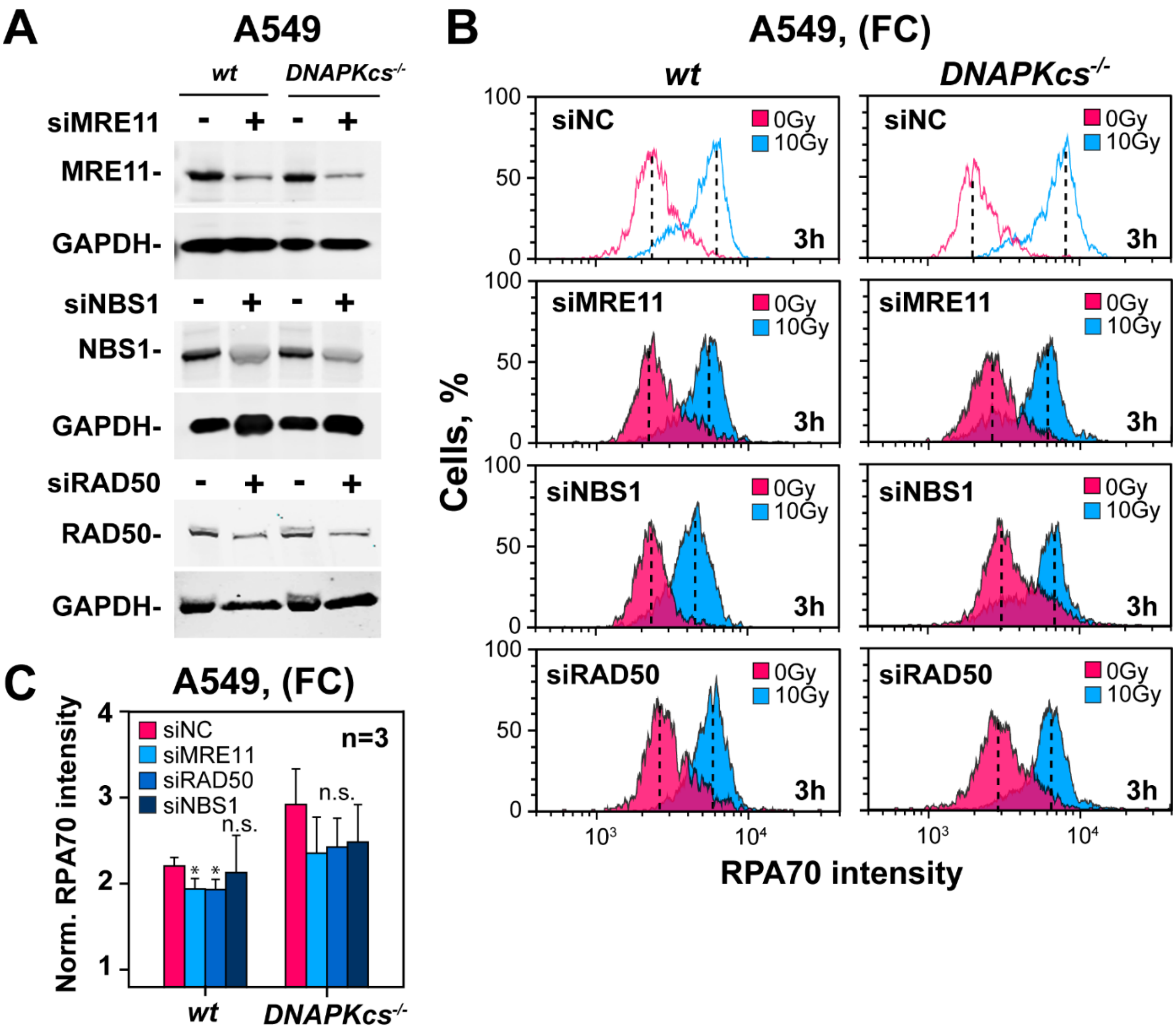

Figure S8. *Resection analysis in wt and DNA-PKcs$^{-/-}$ A549 cells after depletion of components of the MRN complex.* A) Western blot analysis showing the levels of MRE11, NBS1 and RAD50 in *wt* and *DNA-PKcs$^{-/-}$* A549 cells after transfection with the corresponding siRNAs. B) Cell cycle specific FC analysis of RPA70 signal intensity, 3 h after exposure of MRN depleted cells to 10 Gy of IR. C) Quantification of RPA70 signal intensity shown in panel B. The results represent the mean and SE from 3 independent determinations.

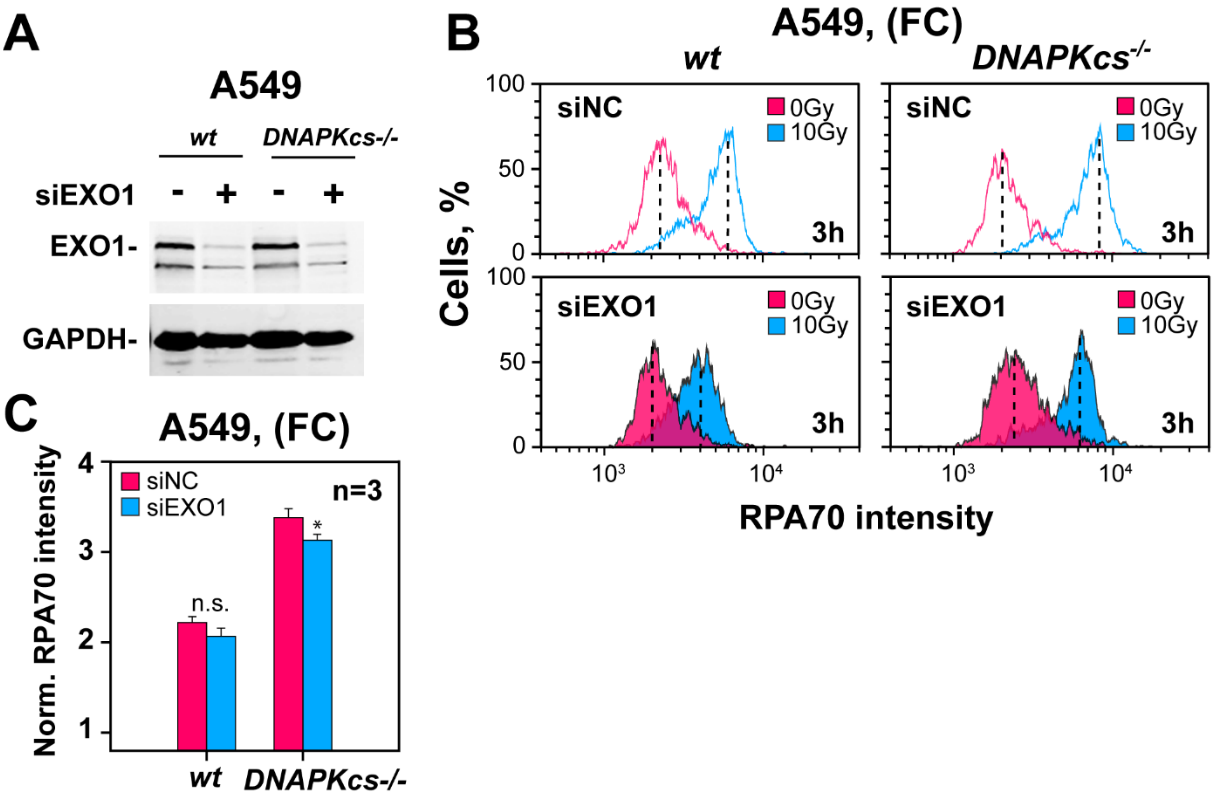

Figure S9. *Effects on resection of EXO1 depletion.* A) Western blot analysis showing the level of EXO1 after transfection with a specific EXO1 siRNAs. B) As in Figure 6B, for cells depleted for EXO1 by transfection with a specific EXO1 siRNA. C) As in Figure 6C, for cells depleted for EXO1.

Table S1. *Sequences of siRNA against human and hamster genes of interests.* For more information see the Materials and Methods.

Target protein	siRNA
**Negative control (siNC)**	1. UUCUCCGAACGUGUCACGU
**EXO1**	1. GCCUUUGCUAAUCCAAUCCCACG
**DNA2**	1. AGACAAGGUUCCAGCGCCA
**CTIP**	1. GCUAAAAC AGGAACGAAUC<br>2. GAUUCGUUCCUGUUUUAGC
**MRE11**	1. GAUGCCAUUGAGGAAUAAG
**RAD50**	1. UAAUGAGACUUGACAAUGA<br>2. ACACUCUUGGGUACAAUAA<br>3. ACAGAACUCCUCACUAAGA
**NBS1**	1. UAACCUUGUUGGCCUGAAGUAGAUG<br>2. CAGGAUGGCUGUCAGGUUA
**BLM**	1. GAGCACAUCUGUAAAUUAA<br>2. CAGGAUGGCUGUCAGGUUA
**DNA-PKcs**	1. CGGCUAACUCGCCAGUUUA
**Human KU80**	1. UUCCUUAAUGGCUUGUCGUUU<br>2. AAACGACAAGCCAUUAAGGAA
**Hamster Ku80**	1. GAAACUGUCUAUUGCUUAA<br>2. CCAUAGGGAAGAAGUUUGA<br>3. GGAUUCCUAUGAGUGUUUA

Table S2. *Statistical analysis of the results shown in main figures.* For more information see the Materials and Methods.

Figure1C	
2Gy, wt vs DNAPKcs $^{-/-}$	P = 0.0013
4Gy, wt vs DNAPKcs $^{-/-}$	P = 0.0026

Figure1E	
10Gy, 1h, wt vs DNAPKcs $^{-/-}$	P = 0.0494
10Gy, 3h, wt vs DNAPKcs $^{-/-}$	P = 0.0495
10Gy, 6h, wt vs DNAPKcs $^{-/-}$	P = 0.0433

Figure2C	
2Gy, CHOK1 vs XR-C1-3	P = 0.0001
4Gy, CHOK1 vs XR-C1-3	P = 0.0001
2Gy, CHOK1 vs V3	P = 0.0003
4Gy, CHOK1 vs V3	P = 0.0003
2Gy, CHOK1 vs Irs20	P = 0.0063
4Gy, CHOK1 vs Irs20	P = 0.0006

Figure2E	
10Gy, 1h, CHOK1 vs XR-C1-3	P < 0.0001
10Gy, 3h, CHOK1 vs XR-C1-3	P = 0.0040
10Gy, 6h, CHOK1 vs XR-C1-3	P = 0.0069
10Gy, 1h, CHOK1 vs Irs20	P = 0.0015
10Gy, 3h, CHOK1 vs Irs20	P = 0.0302
10Gy, 6h, CHOK1 vs Irs20	P = 0.1054

Figure3B	
2Gy, CHO10B4 vs Xrs6	P = 0.7098
4Gy, CHO10B4 vs Xrs6	P = 0.8009

Figure3D	
10Gy, 1h, CHO10B4 vs Xrs6	P = 0.8898
10Gy, 3h, CHO10B4 vs Xrs6	P = 0.8018
10Gy, 6h, CHO10B4 vs Xrs6	P = 0.0717

Figure3G	
10Gy, 1h, siNC vs siKU80	P = 0.5974
10Gy, 3h, siNC vs siKU80	P = 0.3473
10Gy, 6h, siNC vs siKU80	P = 0.5025

Figure4A	
2Gy, wt vs LIG4 $^{-/-}$	P = 0.9986
4Gy, wt vs LIG4 $^{-/-}$	P = 0.1747

Figure4B	
2Gy, wt vs XLF(m)	P = 0.4116
4Gy, wt vs XLF(m)	P = 0.5100

Figure4C	
2Gy, wt vs PAXX $^{-/-}$	P = 0.7155
4Gy, wt vs PAXX $^{-/-}$	P = 0.6523

Figure4E	
CHO10B4 vs V3	P = 0.002
CHO10B4 vs XR1	P = 0.196
CHO10B4 vs Xrs6	P < 0.001

Figure5C	
2Gy, wt vs LIG4 $^{-/-}$	P < 0.0001
4Gy, wt vs LIG4 $^{-/-}$	P = 0.0015

Figure5E	
2Gy, wt vs XLF(m)	P = 0.0006
4Gy, wt vs XLF(m)	P < 0.0001

Figure6C	
wt , siNC vs siDNA2	P = 0.0002
wt , siNC vs siBLM	P = 0.0001
wt , siNC vs siDNA2/siBLM	P = 0.0002
DNAPKcs $^{-/-}$, siNC vs siDNA2	P < 0.0001
DNAPKcs $^{-/-}$, siNC vs siBLM	P < 0.0001
DNAPKcs $^{-/-}$, siNC vs siDNA2/siBLM	P < 0.0001

Figure6E	
wt , untr vs DNA2i	P = 0.0001
DNAPKcs $^{-/-}$, untr vs DNA2i	P < 0.0001

Table S3. *Statistical analysis of the results shown in supplementary figures.* For more information see the Materials and Methods.

FigureS2B
1Gy, untr vs DNAPKcsi
P = 0.0009
4Gy, untr vs DNAPKcsi
P = 0.0266

FigureS2D
A549, untr vs DNAPKcsi
P = 0.3421
M059K, untr vs DNAPKcsi
P = 0.6778

FigureS3E
2Gy, CHO10B4, siNC vs siKu80
P = 0.0686
4Gy, CHO10B4, siNC vs siKu80
P = 0.5339

FigureS3F
2Gy, CHOK1, siNC vs siKu80
P = 0.7089
4Gy, CHOK1, siNC vs siKu80
P = 0.3420

FigureS3G
2Gy, Xrs6, siNC vs siKu80
P = 0.1355
4Gy, Xrs6, siNC vs siKu80
P = 0.2331

FigureS4D
10Gy, 1h, CHO10B4, siNC vs siKu80
P = 0.8095
10Gy, 3h, CHO10B4, siNC vs siKu80
P = 0.0784
10Gy, 6h, CHO10B4, siNC vs siKu80
P = 0.1835

FigureS4E
10Gy, 1h, CHOK1, siNC vs siKu80
P = 0.8012
10Gy, 3h, CHOK1, siNC vs siKu80
P = 0.9130
10Gy, 6h, CHOK1, siNC vs siKu80
P = 0.2703

FigureS4F
10Gy, 1h, Xrs6, siNC vs siKu80
P = 0.9917
10Gy, 3h, Xrs6, siNC vs siKu80
P = 0.9539
10Gy, 6h, Xrs6, siNC vs siKu80
P = 0.7584

Figure5A
2Gy, wt vs LIG4(m)
P = 0.7499
4Gy, wt vs LIG4(m)
P = 0.9483

Figure5B
2Gy, wt vs LIG4 $^{-/-}$
P = 0.9623
4Gy, wt vs LIG4 $^{-/-}$
P = 0.1187

FigureS5D
10Gy, 1h, wt vs LIG4(m)
P = 0.4409
10Gy, 3h, wt vs LIG4(m)
P = 0.9884
10Gy, 6h, wt vs LIG4(m)
P = 0.0737

FigureS5D, HCT116
10Gy, 1h, wt vs LIG4-/-
P = 0.4946
10Gy, 3h, wt vs LIG4-/-
P = 0.4339
10Gy, 6h, wt vs LIG4-/-
P = 0.8338

FigureS5D, HeLa
10Gy, 1h, wt vs LIG4-/-
P = 0.1075
10Gy, 3h, wt vs LIG4-/-
P = 0.0856
10Gy, 6h, wt vs LIG4-/-
P = 0.1208

FigureS6A
10Gy, 1h, wt vs XLF(m)
P = 0.1494
10Gy, 3h, wt vs XLF(m))
P = 0.5396
10Gy, 6h, wt vs XLF(m)
P = 0.3477

FigureS6B
10Gy, 1h, wt vs PAXX $^{-/-}$
P = 0.8723
10Gy, 3h, wt vs PAXX $^{-/-}$
P = 0.6840
10Gy, 6h, wt vs PAXX $^{-/-}$
P = 0.9533

FigureS6C
10Gy, 1h, wt vs XRCC4(m)
P = 0.8898
10Gy, 3h, wt vs XRCC4(m)
P = 0.7875
10Gy, 6h, wt vs XRCC4(m)
P = 0.0717

FigureS7B
2Gy, wt vs Artemis(m)
P = 0.8659
4Gy, wt vs Artemis(m)
P = 0.0718

FigureS7D
10Gy, 1h, wt vs Artemis(m)
P = 0.4946
10Gy, 3h, wt vs Artemis(m)
P = 0.4339
10Gy, 6h, wt vs Artemis(m)
P = 0.8338

FigureS8C
A549, wt , siNC vs siMRE11
P = 0.0426
A549, wt , siNC vs siNBS1
P = 0.0370
A549, wt , siNC vs siRAD50
P = 0.7767
A549, DNAPKcs $^{-/-}$, siNC vs siMRE11
P = 0.1711
A549, DNAPKcs $^{-/-}$, siNC vs siNBS1
P = 0.1810
A549, DNAPKcs $^{-/-}$, siNC vs siRAD50
P = 0.2751

FigureS9B
A549, wt , siNC vs siEXO1
P = 0.0773
A549, DNAPKcs $^{-/-}$, siNC vs siEXO1
P = 0.0773
